# Neurobiological correlates of obsessive‐compulsive disorder (OCD): A narrative review

**DOI:** 10.1002/ibra.70013

**Published:** 2026-01-25

**Authors:** Giulio Perrotta, Anna Sara Liberati

**Affiliations:** ^1^ Department of Clinical Psychology, Faculty of Psychology Universitas Mercatorum Rome Italy; ^2^ Department of Neurosciences, Faculty of Psychology Università Telematica Internazionale “Uninettuno” Rome Italy

**Keywords:** cortico‐striato‐thalamo‐cortical circuit, limbic system, neuroanatomical correlates, obsessive‐compulsive disorder, prefrontal cortex

## Abstract

Obsessive‐compulsive disorder (OCD) is a common and disabling, as well as underdiagnosed, neuropsychiatric condition characterized by involuntary and unwanted obsessions and/or compulsions, often accompanied by states of severe anxiety, distress and shame, as well as other comorbid disorders. Despite the extensive literature available to date, only some of the neurobiological mechanisms underlying the symptomatic manifestations of the disorder have been clarified, underlining the need for further research. The brain structures involved are hippocampus, amygdala, striatum, thalamus, dorsolateral prefrontal cortex, anterior cingulate cortex, and orbitofrontal cortex; furthermore, most studies have mainly focused on the expressive modalities and on the individual structural and functional alterations of the brain, generating sometimes conflicting data. The aim of this article is therefore to summarize and bring together the main evidence collected so far on what would appear to be the neuroanatomical correlates and mechanisms underlying the disorder and its manifestations, to provide a sufficiently clear and complete overview.

## Graphical abstract

Obsessive‐compulsive disorder (OCD) is a common and disabling, as well as underdiagnosed, neuropsychiatric condition characterized by involuntary and unwanted obsessions and/or compulsions often accompanied by states of severe anxiety, distress and shame, as well as other comorbid disorders. Despite the extensive literature available to date, only some of the neurobiological mechanisms underlying the symptomatic manifestations of the disorder have been clarified, underlining the need for further research. Furthermore, most studies have mainly focused on the expressive modalities and on the individual structural and functional alterations of the brain, generating sometimes conflicting data.

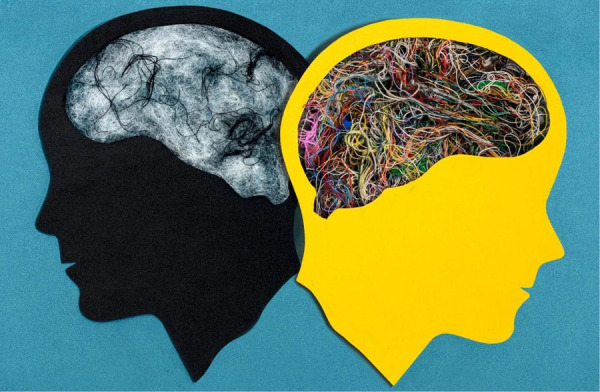

## 1 INTRODUCTION

The Diagnostic and Statistical Manual of Mental Disorders, Fifth Edition Revised (DSM‐V‐TR) defines obsessive‐compulsive disorder (OCD) as a pervasive and disabling disorder, characterized by obsessions and compulsions “severe enough to consume time, cause significant distress, or significantly impair behavior”,^1^ significantly interfering with the person's normal functions and activities, including educational, occupational, social and relational ones. Recent estimates of the incidence of this disorder report a diagnosis rate of about 4% compared to the total general population, thus highlighting its pervasiveness.^2^ Furthermore, in general, most of these patients present both obsessive thoughts and compulsive behaviors together. Despite the relatively recent recognition of OCD as a pathology, and it is no longer included among anxiety disorders as it has its own diagnostic specificity, clinical and experimental data are increasingly highlighting its symptomatic, neurobiological, neuropsychological and genetic heterogeneity, as well as in the individual response to treatment,^3^ somewhat complicating the clinical‐diagnostic picture.

Research conducted on identical and fraternal twins, but also on first‐ and second‐degree relatives, has in fact demonstrated that OCD is a multifactorial familial condition involving both polygenic, neurobiological and environmental risk factors.^4^ For example, several neuroimaging studies have highlighted the involvement of the cortico‐striato‐thalamo‐cortical (CSTC) circuit in the physiopathology of the disorder,^5^ also supported by the observation of the presence of specific neuropsychological impairments in OCD patients, mainly attributable to executive functions. Genetic studies, for their part, indicate that it is above all the genes that influence the serotonergic, dopaminergic, glutamatergic systems—and their interactions—that play a crucial role in the functioning of this circuit. In this regard, a 2017 study from the Broad Institute in Cambridge identified four genes that have a high probability of playing a causal role in the genesis of OCD.^6^ These are some variants of the neurexin 1 (*NRXN1*), 5‐hydroxytryptamine receptor 2A (*HTR2A*), cortactin binding protein 2 (*CTTNBP2*) gene and the receptor accessory protein 3 (*REEP3*) that regulate the behavior of cellular vesicles in which neurotransmitters accumulate to be released into the synaptic cleft. Regarding environmental factors, it has been observed that early adverse events, psychological trauma and neurological disorders can modify the expression of gene clusters linked to the risk of evolving the pathology, thus facilitating the manifestation of obsessive‐compulsive behaviors.^7^ In addition, several studies evaluated the link between bacterial infections and the development of OCD symptoms and tics. Specifically, it has been hypothesized that streptococcal infection may cause, in predisposed individuals, an anomalous production of autoantibodies that bind to nerve cells, particularly the basal ganglia, and it is associated with alterations in the structures that are part of it. The term “Pediatric Autoimmune Neuropsychiatric Disorders Associated with Streptococcal infections” (PANDAS), for example, refers to a group of patients in whom the onset or worsening of OCD symptoms or tics occurs suddenly following streptococcal bacterial infections.^8^ Neuroimaging studies conducted on these patients have highlighted anomalous volumetric increases, specifically at the level of the basal ganglia. The involvement of the immune system has also been highlighted by quantitative alterations of the inflammatory cytokines TNF‐alpha, IL‐6 and IL‐1, detected in the serum of patients with OCD and Tourette syndrome. A 2020 study instead found high levels of the Imood protein in OCD patients,^9^ an immunomodulin believed to play a key role in regulating anxious behavior so that, according to the evidence, it would appear to be a good marker for this disorder as well as an interesting target for treatment. Against this background, the multifactorial nature of the etiopathogenesis of OCD is certainly indisputable, so, consequently, the importance of organizing the mass of neurobiological data relating to the disorder itself to facilitate its consultation by those who study it strongly emerges.

## 2 AIM AND METHODOLOGY

The aim of this article is therefore to summarize and bring together the main evidence collected so far on what would appear to be the neuroanatomical correlates and mechanisms underlying the disorder and its manifestations, to provide a sufficiently clear and complete overview.

The bibliographic search was conducted on PubMed, Scopus and Google Scholar, covering studies published up to January 2025, without any linguistic limitation. Inclusion criteria were: (1) neural correlates of OCD and (2) systematic review, meta‐analysis, and research manuscripts.

## 3 DESCRIPTIVE, CLINICAL AND DIAGNOSTIC ELEMENTS OF OCD

The main and most evident symptomatic and diagnostic characteristic of OCD is the presence of obsessions and compulsions. Obsessions are phobic thoughts, mental images or repetitive, unwanted and intrusive impulses, mostly ego‐dystonic, which can cause a state of severe anxiety or anguish and, consequently, the implementation of avoidance behaviors towards situations or events experienced as uncomfortable, with consequent significant reduction in social interactions and quality of life in general. Typical obsessive behaviors can be, for example: excessive personal and/or environmental hygiene linked to phobias of contamination; the repetitive performance of ritual and/or superstitious acts in the belief that, by doing so, one can exercise a certain control over events; an exasperated concern for one's own and/or others' safety that manifests itself through ritual and often invasive actions, aimed at maintaining/controlling it; a particular attention to the symmetry and order of environments and objects, almost always accompanied by the uncontrollable impulse to catalogue, rearrange or count the elements, and so forth. Compulsions, on the other hand, are ritual and repetitive behaviors or mental acts that are performed in response to an obsession in order to reduce the resulting anxiety/anguish or to prevent a real or, more often, imaginary, feared consequence.^10^ It is no coincidence that the term itself derives from the Latin “compellere” which means “to force, to constrain,” referring precisely to the concept of “obligation to act.” Statistical data demonstrate how both these types of behavior are expressed in a similar way by patients who are very different in age, sex and culture of origin, thus suggesting a high degree of commonality in the symptomatic dimensions of the disorder itself.^11^ Often, OCD symptoms appear already in adolescence but in women it is not uncommon for them to arise (or, in patients already diagnosed, exacerbate) in the perinatal period, mainly due to the pressure and psychological vulnerability to which new mothers are often subjected.^12^

The diagnosis is complicated both by the fact that similar symptoms can also be found in other disorders, such as phobias, generalized anxiety disorder, tic disorders and mania, and by the comorbidities often present in these patients. Therefore, it is of fundamental importance to carefully evaluate the characteristics and specific manifestations, as well as the degree of intrusiveness in the functioning and daily life of the patients themselves, according to internationally shared diagnostic criteria, such as the DSM‐V‐TR which separates OCD and related disorders from anxiety disorders, in relation to the lack of response to benzodiazepines and the hypothesis that the origin of obsessions and compulsions is a dysfunction in the circuits that go from the frontal cortex to the basal ganglia. In this sense, OCD is now considered more as an expression of an altered coding of gratification rather than as a condition of anxious alarm. The diagnostic category of OCD and related disorders includes: (1) obsessive‐compulsive disorder; (2) body dysmorphic disorder; (3) trichotillomania; (4) excoriation disorder; (5) hoarding disorder; (6) substance‐ or medication‐induced OCD; (7) other specific or unspecified OCD (e.g., body‐focused repetitive behavior, obsessive jealousy).

Currently, the diagnosis of OCD is predominantly clinical, based on the identification, during an interview, of the symptoms and signs just described. In fact, there are still no diagnostic biomarkers that support it in a precise and univocal way. However, several neuroimaging and morphometric investigations^13^, ^14^ are providing evidence of the existence, in these patients, of recurrent anomalies affecting some cortical and subcortical neural circuits, as well as the data support the hypothesis that there are correlations with widespread alterations in the limbic, parietal and cerebellar regions. Additionally, a recent study by researchers at Baylor College of Medicine and Texas Children's Hospital identified a prominent, highly predictable circadian pattern of electrical activity (theta/alpha power, 9 Hz) in the ventral striatum of patients with severe OCD that appears to have the potential to predict and monitor patients' clinical status and specifically guide deep brain stimulation (DBS) therapy, with promising results.^15^

## 4 THE MAIN NEUROANATOMICAL AND FUNCTIONAL CORRELATES OF OCD

In recent years, both structural and functional neuroimaging studies have provided a solid contribution to the understanding of the neural abnormalities underlying OCD. In particular, the role of the CSTC circuit has been highlighted: a highly integrated network of cortical and subcortical structures that collectively manage and regulate sensorimotor, limbic, and cognitive functions. In fact, this circuit regulates and controls both the expression of goal‐oriented behaviors and the emotional, cognitive, and motivational processes that guide them. For example, ventral structures, such as the nucleus accumbens (NA), the olfactory tubercle (OT), and the mesencephalic nuclei, play a key role in reinforcement and reward and are important in the development of habits and addictive behaviors.^16^ While others, such as the striatum, globus pallidus (GP), and thalamus, are involved not only in motor functions but also in more cognitive functions, such as procedural learning and working memory.^17^, ^18^ The correct functionality of the CSTC is guaranteed by the coordinated interaction between two different populations of long‐projecting GABAergic neurons (expressing different dopamine receptors, D1 or D2) that act in parallel and opposite ways to each other, each managing a specific transmission pathway. Thus, we can distinguish: a “direct” (excitatory) pathway projecting from the orbitofrontal cortex (OFC) and the cingulate gyrus to the medial portion of the striatum, which includes the caudate nuclei and the putamen. Inhibitory fibers directed both to the internal globus pallidus (GPi) and to the pars reticulata of the substantia nigra (SNr) originate from D1 neurons of the putamen. In this way, GPi neurons cannot inhibit the anterior thalamic nuclei, to which they in turn project, thus facilitating the transmission of the impulse directed to the cortex. For this reason, this pathway is also known as the “fast pathway”. In contrast, in the “indirect” (inhibitory) pathway, GABAergic fibers are projected from D2 neurons in the putamen to the external portion of the globus pallidus (GPe), preventing it from inhibiting the subthalamic nucleus, which is then activated, sending excitatory glutamatergic impulses to the GPi. This causes thalamic inhibition and, therefore, a slowing of nerve transmission. Under normal conditions, these two pathways are in perfect balance with each other, acting as “biochemical traffic lights” that manage the activation and inhibition of impulses according to needs. However, there is some evidence to support the hypothesis that the symptomatic manifestations of OCD may be due to a lack of coordination between these two “traffic lights” with an imbalance in favor of the excitatory pathway compatible with the aberrant behavioral manifestations typical of the disorder itself.^19^ In fact, the thalamus, the sensory and motor sorting center, is hyperactive in OCD precisely because of the lack of inhibition by the caudate nucleus.^20^ This condition amplifies the flow of signals towards the OFC and the anterior cingulate, contributing to the maintenance of the obsessive‐compulsive cycle. Further supporting this consideration is the demonstration that, by repeatedly stimulating the cortico‐striatal glutamatergic afferents—and therefore favoring the “excitatory pathway”—behaviors like those manifested in OCD are triggered in mouse models. While, on the contrary, the stimulation of the feed‐forward inhibition mechanism—which activates the inhibitory pathway ‐ alleviates them.^21^, ^22^

**Figure 1:**
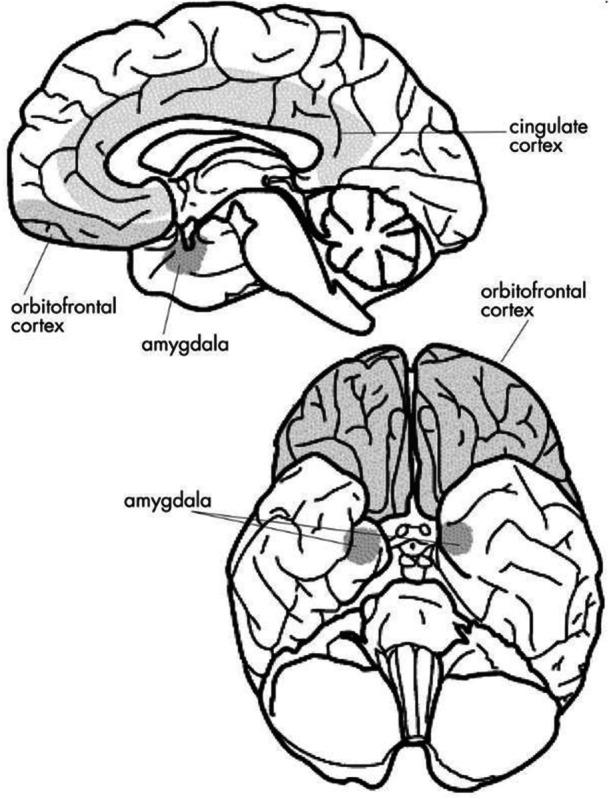
The key brain areas implicated in OCD. Reprinted from Huey et al., *J Neuropsychiatry Clin Neurosci*. 2008; 20(4):390‐408.^20^

Regarding the OFC (Figure 1), there is evidence in the literature to support its direct involvement in the etiopathogenesis of OCD, mainly related to its inability to properly regulate the cortico‐striatal circuits involved in decision‐making processes and threat assessment. Under physiological conditions, the OFC works in concert with other crucial areas (including, in addition to the basal ganglia, the ACC, the amygdala, and the hippocampus) to modulate behavioral responses to relevant stimuli. This function depends on a delicate balance between processing emotional information and the ability to inhibit inappropriate, unnecessary, or excessive behavioral responses to the situation. Therefore, when this balance is disturbed, it can contribute to the emergence of obsessive thoughts and compulsive behaviors typical of OCD. For example, functional magnetic resonance imaging (fMRI) and positron emission tomography (PET) scans have revealed consistent hyperactivity of the OFC in subjects with OCD compared to healthy controls. This hyperactivity was particularly evident when subjects were exposed to stimuli that evoked obsessions or were related to compulsive rituals. A meta‐analysis highlighted a marked hyperactivity of the OFC in OCD patients compared to healthy controls, with a predominant involvement of the ventral orbitofrontal circuit (vOFC) that seems to be closely linked to the persistence of obsessive thoughts.^23^ This phenomenon seems to reflect the inability of the OFC to adequately regulate danger signals, maintaining the system in a state of “chronic alert.” From a structural point of view, volumetric studies conducted with MRI have shown an increase in gray matter density in the OFC in some patients with OCD, suggesting a potential compensatory mechanism or intrinsic neuroanatomical dysfunction. In parallel, reduced connectivity between the OFC and other critical regions such as the thalamus and the caudate nucleus has been observed.^24^ This alteration in functional connectivity could explain the patient's difficulty in modulating behavioral responses, contributing to the cognitive rigidity and repetitiveness that characterize the disorder. An additional noteworthy element in the pathogenesis of OCD is the dysfunction of the fronto‐striatal circuits, a set of neural pathways that connect the OFC to the basal ganglia (in particular, the caudate nucleus and the putamen) and the thalamus and that play an essential role in filtering relevant information and modulating behavior based on priorities. In OCD patients, hyperconnectivity is observed between the OFC and the caudate nucleus with an increase in the grey matter volume in the lenticular nucleus, and caudate while smaller grey matter volume in frontal eye fields, which leads to excessive processing of intrusive stimuli and difficulty inhibiting inappropriate responses.^25^, ^26^ This dysfunction can therefore be compared to a “stuck circuit,” in which alarm signals are continuously reinforced without adequate resolution.

The anterior cingulate cortex (ACC) is involved in numerous cognitive and affective functions, such as error monitoring and detection of the presence of cognitive conflict and is also particularly relevant to the psychopathology of OCD. In several studies conducted through neuroimaging and detection of event‐related potentials, in fact, a significant hyperactivation of this region has been detected, together with reduced volumes of the gray matter but not of the white matter.^27^, ^28^ These alterations manifest themselves as a persistent feeling of incompleteness or dissatisfaction, fueling the need to carry out compulsions. A study conducted through task‐induced functional connectivity analysis, revealed the presence of a strong connectivity between the ACC and the dorsolateral prefrontal cortex (dlPFC) in OCD patients during the execution of the Stroop task,^29^ supporting the hypothesis of an anomalous error processing and a cortico‐cortical interaction that can negatively influence the decision‐making process. Other studies, conducted on OCD patients who have suffered a lesion of the ACC—particularly in its dorsal portion (dACC)—(e.g., following anterior cingulotomy) have shown that this condition is often associated with a significant reduction in symptom severity, especially in cases of severe disease refractory to conventional treatments.^30^ In parallel, the therapeutic success of selective serotonin reuptake inhibitors (SSRIs) in OCD has been associated with a reduction in dACC metabolism.^31^ These data reinforce the idea that hyperactivation of the dACC may contribute to the generation of aberrant error signals that fuel obsessions, as well as indicating a possible role, together with the amygdala, in the excessive responses to fear and anxiety typical of patients with OCD.

**Figure 2:**
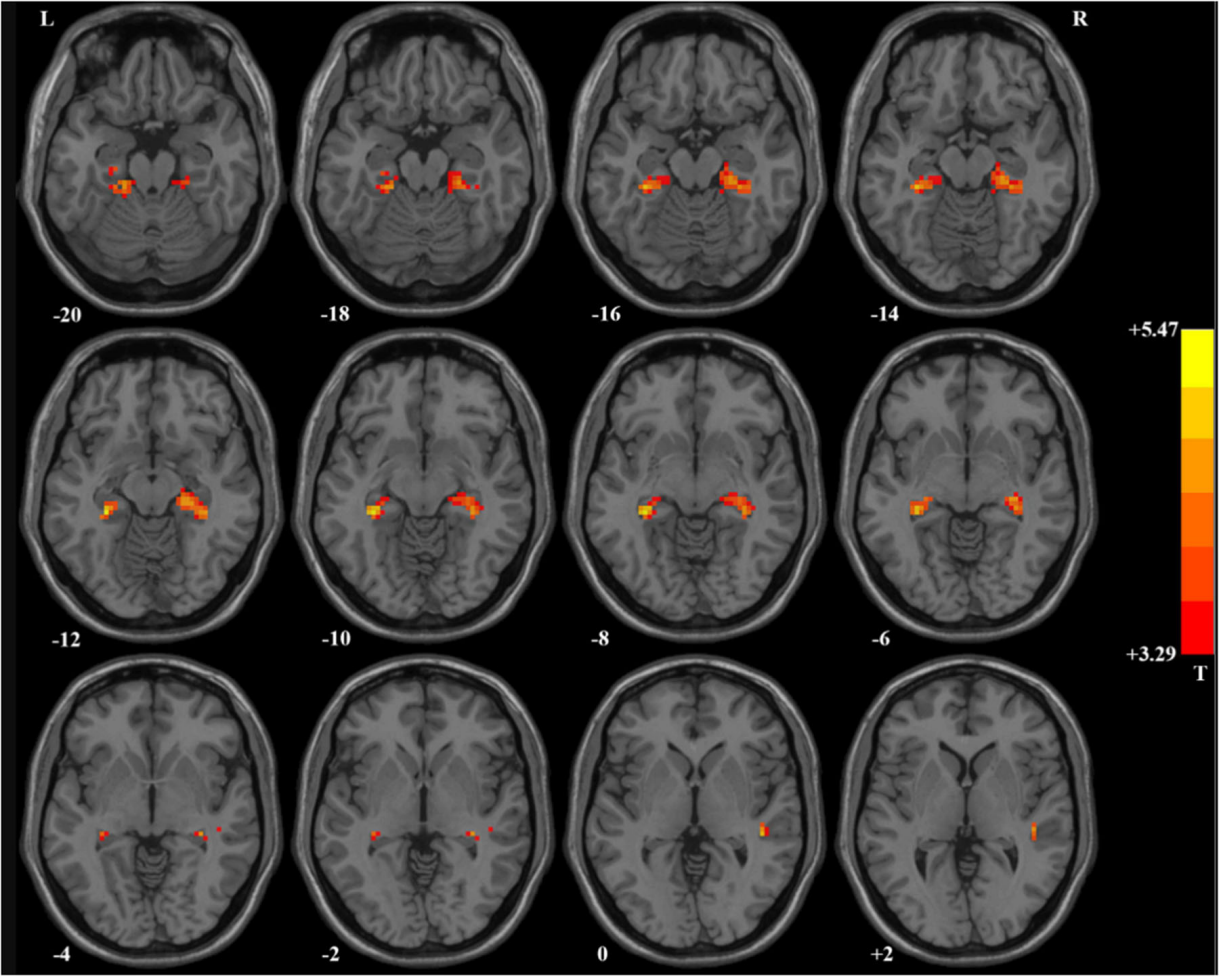
Abnormal spontaneous neural activity in hippocampal–cortical system of patients with obsessive–compulsive disorder. Compared with healthy controls, patients exhibited increased regional homogeneity (ReHo) values in the right superior temporal gyrus as well as in the bilateral hippocampus, parahippocampus, fusiform gyrus, and cerebellum. Reprinted from Yan et al., *Front Cell Neurosci*. 2022;16:906534.^32^

In this regard, the limbic system and its component structures—particularly the hippocampus and amygdala (Figure 2)—are also morphologically and functionally altered in OCD. While the amygdala contributes to the anxiety component of the disorder, the hippocampus shows alterations in the ability to process memories that reduce obsessions. Therefore, although obsessions and compulsions are not a direct product of emotional dysregulation, the general idea is that these dysregulations, like pathological anxiety, still have some relevance to the etiopathology of OCD, even if it is not yet fully understood. PET and fMRI studies have, for example, reported a hyperactivity of the amygdala in response to stimuli related to obsessions,^33^ suggesting a possible hypersensitivity to threatening or aversive stimuli. This is in fact compatible with the idea that abnormalities in the amygdala strongly contribute to the intense experience of anxiety that characterizes the disorder itself. Other studies, however, have observed significant volumetric reductions of the amygdala in OCD patients compared to healthy controls,^34^ suggesting instead an inability of the amygdala to filter and discriminate emotional triggering stimuli. A third line of investigation, finally, highlights that, although exposure to specific OCD‐related stimuli (e.g., phobias) has often been associated with exaggerated amygdala activity in these patients, some studies have reported a hypoactivation of the amygdala compared to healthy controls in response to emotionally impactful but non‐specific stimuli (e.g., familiar faces),^35^, ^36^ advancing the interesting hypothesis of a “stimulus‐selective activation” of the amygdala in OCD. Consequently, it is plausible to suggest that obsessive and compulsive behaviors may be the result of disproportionate emotional representations. So, for example, the mere sight of a household cleaning product might stimulate compulsions for hygiene and cleanliness. This may also help explain why individual OCD patients suffer from specific and “personalized” obsessions, rather than generic ones. That is why each individual patient tends to be obsessed with only a small subset of the vast array of possible obsessions, rather than all of them. As regards the hippocampus, although there is not yet a full consensus on the presence, in OCD, of specific anomalies affecting this structure and the areas associated with it, some studies have documented the presence of a volumetric reduction affecting in particular: subiculum, presubiculum and hippocampal tail—more accentuated in the right hippocampus—and of bilateral deformities in patients compared to control subjects.^37^ Among these, a research highlighted in subjects with OCD the presence of a characteristic “downward” displacement of the hippocampal head, compared to healthy subjects.^38^ It is also interesting to note that, since in several stress‐related psychiatric disorders (such as depression and PTSD) the hippocampal volume is lower than normal^39^ and, since patients with OCD tend, in general, to report high levels of stress and anxiety, it is not unlikely to suppose that notable morphological and functional differences of the hippocampus may also exist in OCD. In this regard, a study aimed at investigating the possible relationship between hippocampal dimensions and symptomatic profiles of OCD,^40^ highlighted how, with greater severity of symptoms, smaller volumes of this region corresponded with respect to both patients with milder symptoms and normal control subjects, suggesting a possible association between structural alterations of the hippocampus and severity of symptoms and behaviors manifested.

## 5 DISCUSSION

OCD is undoubtedly a complex neuropsychiatric condition, characterized by considerable heterogeneity both at the symptomatic and neurobiological levels (Table 1). The presence of obsessions and compulsions represents the diagnostic core of the disorder, determining a significant negative impact on the quality of life of patients.^41^ Neuroimaging studies and genetic investigations have highlighted the crucial role of the CSTC circuit, and the neurotransmitter systems associated with it, such as serotonergic, dopaminergic and glutamatergic ones. CSTC is a highly integrated network of cortical and subcortical structures that regulate sensorimotor, limbic, and cognitive functions. The lack of coordination between the direct (excitatory) and indirect (inhibitory) pathways within this circuit, with an imbalance in favor of the excitatory pathway, is thought to be responsible for the symptomatic manifestations of OCD. Hyperactivity of the thalamus due to the lack of inhibition by the caudate nucleus amplifies the flow of signals to the OFC and the ACC, contributing to the maintenance of the obsessive‐compulsive cycle. The OFC plays a crucial role in regulating the cortico‐striatal circuits involved in decision‐making and threat assessment. OFC hyperactivity and reduced functional connectivity with the thalamus and caudate nucleus thus contribute to the persistence of obsessive thoughts and compulsive behaviors, keeping the system in a state of “chronic alert.” The ACC, involved in error monitoring and cognitive conflict detection, also shows hyperactivity and reduced gray matter volumes in OCD patients, fueling the need to perform compulsions to relieve the feeling of incompleteness. The limbic system, and in particular the amygdala and hippocampus, are also morphologically and functionally altered in OCD. Hyperactivity of the amygdala in response to obsession‐related stimuli suggests possible hypersensitivity to threatening stimuli, contributing to the experience of intense anxiety typical of the disorder. The hippocampus, on the other hand, shows correlations with alterations in the ability to process memories that reduce obsessions, suggesting a possible link between structural alterations of the hippocampus and the severity of symptoms. Finally, the multifactorial nature of the etiopathogenesis of OCD has been further supported by the identification of genetic variants affecting the *NRXN1*, *HTR2A*, *CTTNBP2*, and *REEP3* genes, as well as by the significant role of environmental factors, such as early adverse events and bacterial infections. All these elements can therefore contribute to shaping the expression of genes linked to the risk of developing the disorder and to facilitate the manifestation of obsessive‐compulsive symptoms. Recent discoveries in the field of neuroimaging, such as the identification of circadian patterns of electrical activity in the ventral striatum, open new perspectives for the monitoring and targeted treatment of the disorder through innovative techniques such as DBS, repetitive transcranial magnetic stimulation (rTMS) and transcranial direct current stimulation (tDCS). However, some limitations still remain. In particular, the fact that most morphological and functional investigation studies have been conducted on single sites with relatively small sample sizes, which leads to results that are not always replicable. This variability could depend on the heterogeneity of the selected samples, differences in imaging protocols, or both. Second, although it is hypothesized that specific brain abnormalities underlie dysfunctions in certain neural processes, which in turn give rise to OCD symptoms, there is still a lack of clear correlation between these abnormalities, alterations in neural processes, and the characteristic clinical profiles of the disorder. Finally, it is important to emphasize that most studies are of a correlative nature and, consequently, do not allow to establish whether the observed brain abnormalities are the cause of OCD symptoms or rather a consequence of them.

**Table 1:** Neuroanatomical and functional differences between a healthy subject and a subject with OCD.

Neuroanatomical areas	Healthy subject (Average adult)	Person with OCD
Hippocampus	Bilateral medial temporal lobe fold of mean length ≈8 cm. Diffusely innervated by afferent and efferent fibers to other CNS structures. Principal Center for Memory and Learning also handles functions of spatial orientation, and perceptual processing, object recognition, socioemotional info processing and subsequent behavioral responses, and stress management.	Involved in memory and in the contextualization of experiences, it can be altered in obsessive rumination. Also, volumetric reductions have been documented in the subiculum, presubiculum and hippocampal tail—more accentuated in the right hippocampus—and bilateral deformities, thus suggesting a possible symptom‐related effect.
Amygdala	Placed bilaterally in the anterior portion of each of the medial temporal lobes. Reaches an average vol. of ≈2.30 ± 10 cm^3^ (larger on the right), larger in males. It consists of 13 distinct nuclei, each with its functions and connections to other brain structures. Overall, it participates in the processes of emotional and olfactory memory, processing of sensory info, management of emotions—particularly anger and fear—and behavioral, neurovegetative and hormonal responses to them.	Regulates emotional and fear responses. An altered interaction between the amygdala and the orbitofrontal cortex may contribute to the anxious component of OCD. Also, studies documented hyperactivity of the amygdala in response to obsession‐related stimuli, suggesting a possible hypersensitivity to threatening or aversive stimuli
Striatum (corpus striatum) (Nuclei Caudate and Putamen)	Cluster of interconnected nuclei that make up the largest structure of the subcortical basal ganglia. It acts as a filter for signals that pass from the cortex to the thalamus, inhibiting those that are not relevant and allowing only the necessary information to pass through.	Inhibitory dysfunction, especially at the level of the caudate nucleus, causes an excess of thalamic signals to the cortex. This uncontrolled flow can contribute to the persistence of obsessive thoughts and the need for compulsive actions.
Thalamus	Composed of about 50 nuclei, including the medial and lateral geniculate bodies, it integrates and transmits sensory information, participates in the control of movements and memory. It is the “electrical panel” of the cerebral cortex, which allows global or selective hemispheric activation. It is divided into four parts: Anterior part, Medial part, Lateral part, and Posterior part.	Excessive stimulation due to a lack of inhibition by the caudate nucleus. The thalamus amplifies obsessive signals, reinforcing the pathological circuit.
Dorsolateral Prefrontal Cortex (DLPFC)	Critically involved in cognitive control, particularly in overriding emotional biases and making complex social decisions by balancing emotional and deliberative processes. It also supports executive functions, such as cognitive control, planning, and behavior regulation.	Impaired connectivity with the cortico‐striato‐thalamo‐cortical circuit (CSTC), which impairs the ability to interrupt obsessive thoughts and compulsive behaviors.
Anterior cingulate cortex (ACC)	Given the direct connections it establishes with the prefrontal cortex (PFC) and some limbic structures (amygdala, hypothalamus, and hippocampus), it participates in the encoding of emotions, particularly anxiety, anger, and fear, and regulates some endocrine and vegetative functions.	Often overactive, resulting in a persistent feeling of “error” or “incompleteness.” This manifests clinically as the need to perform repetitive compulsions to face and resolve a situation perceived as erroneous, malicious, or abnormal.
Orbito Frontal Cortex (OFC)	Involved in the evaluation of the consequences of actions, in the control of decisions and in emotional regulation. It is crucial for identifying significant or potentially risky stimuli.	Chronic hyperactivity leads to a biased assessment of risks and an increase in the perception of threat. This can explain the onset of obsessions, as stimuli are mistakenly labeled as dangerous or unresolved.

### 5.1 Limitations and future directions

The main strengths of the narrative manuscript are the comprehensive structure of the content focusing on the neurobiological basis of OCD, using a wide literature. Different perspectives from structural, functional and molecular neuroscience are combined, focusing in particular on the CSTC circuit, which is fundamental to understanding how OCD works. It also addresses modern treatment ideas such as DBS, rTMS and biomarker development. The weaknesses are instead related to the narrative choice of the structure, rather than operating with a systematic or meta‐analysis. Future research should therefore continue to explore the neurobiological mechanisms underlying OCD, to improve the clinical management and quality of life of patients affected by this disorder.

## 6 CONCLUSIONS

The integrated understanding of genetic, neurobiological and environmental factors undoubtedly represents a promising path to outline more accurate diagnostic frameworks and develop more effective therapeutic interventions. The literature shows adequate correlations between OCD and structural and functional alterations of hippocampus, amygdala, striatum, thalamus, dorsolateral prefrontal cortex, ACC, and OFC.

## AUTHOR CONTRIBUTIONS

Giulio Perrotta conceived the overall conceptual framework of the review and supervised the development of the manuscript. Anna Sara Liberati conducted the literature review, synthesized the evidence, and drafted the first manuscript. Giulio Perrotta critically revised the manuscript for important intellectual content. All authors have read and approved the final version of the manuscript.

## CONFLICT OF INTEREST STATEMENT

The authors declare no conflicts of interest.

## ETHICS STATEMENT

These authors have nothing to report.

## Data Availability

Not applicable as no new data is generated in this review article.
